# *HSFA1d* regulates the kinetics of heat-induced *HSP17.6* expression in Arabidopsis

**DOI:** 10.17912/micropub.biology.000555

**Published:** 2022-04-15

**Authors:** Erin J Kast, Nick Kaplinsky

**Affiliations:** 1 Department of Biology, Swarthmore College

## Abstract

Arabidopsis contains four *HSFA1* heat shock transcription factors ( *HSFA1a* , *HSFA1b* , *HSFA1d* , and *HSFA1e* ) that regulate the primary response to high temperature stress responses. These genes have overlapping functions and, while double and triple *HSFA1* mutants have thermotolerance phenotypes, these genes have no reported single mutant thermotolerance phenotypes. We used an automated fluorescence microscopy system to quantitate the expression of a *HSP17.* 6:GFP reporter with high temporal resolution to show that *HSFA1d* is required for normal heat-induced *HSP17.6* expression. *HSP17.6* expression is reduced and delayed in *hsfa1d-1* mutants. This finding highlights the power of using gene expression kinetics as a quantitative phenotype for discovering the function of genes that exhibit functional redundancy.

**Figure 1.**
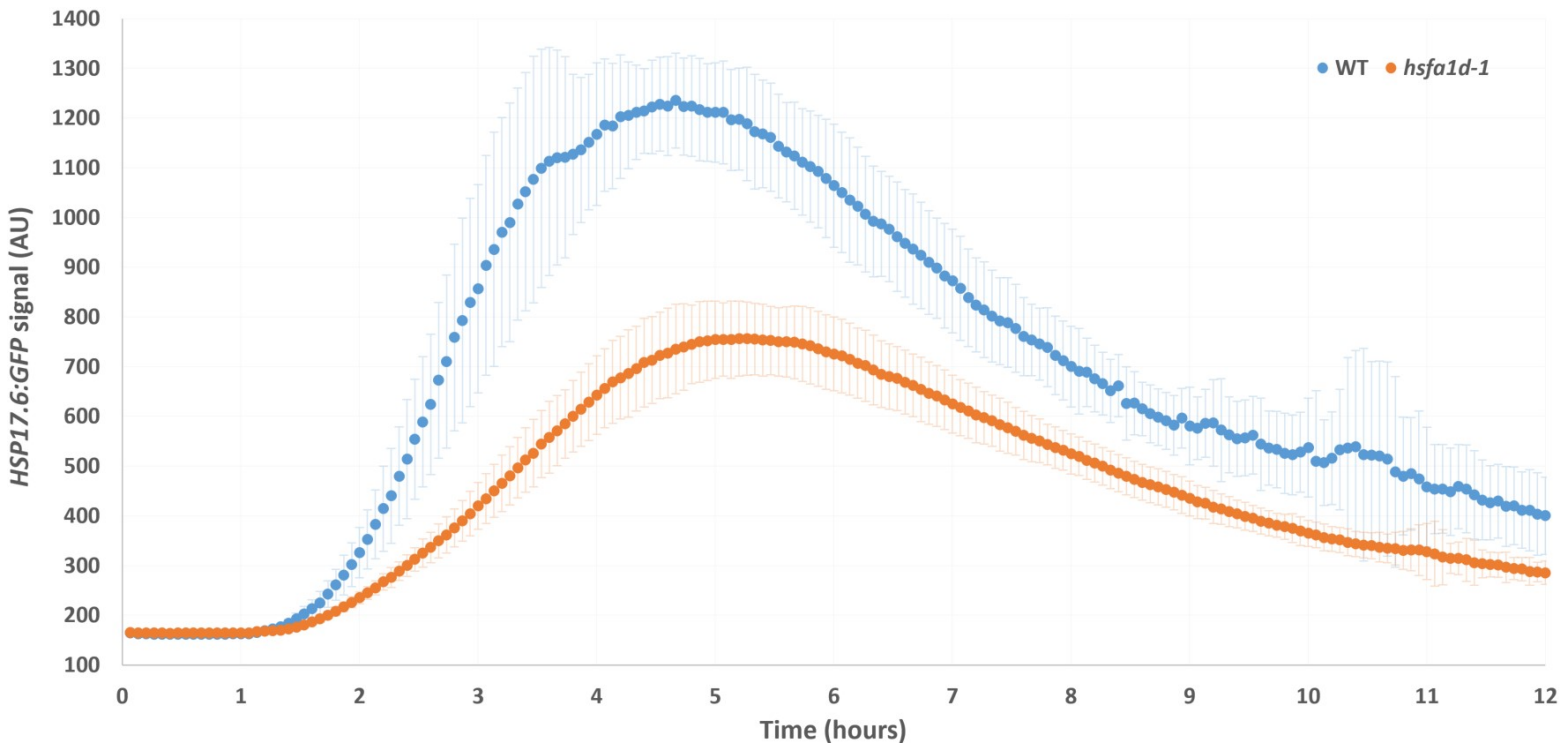
*HSP17.6p:GFP* heat shock kinetics in wild type and *hsfa1d-1* seedlings. Four day old seedlings were heat shocked for one hour at 37 ^o^ C starting at time point 0. They were grown for an additional 11 hours at 22 ^0^ C after the heat shock and images of their roots were acquired every four minutes both during and after the heat shock. Wild type plants (n=11) are shown in blue, *hsfa1d-1* plants (n=49) in orange. *Hsp17.6:GFP* expression is measured in arbitrary units. Error bars are +/- S.D.

## Description

In plants, phenotyping the heat shock response (HSR) is often performed using thermotolerance assays based on survival or germination or by measuring RNA or protein levels at a single or a small number of time points (Yeh et al., 2012; Silva-Correia et al., 2014). These approaches have a limited ability to detect HSR phenotypes in genes with quantitative effects such as members of gene families with partially overlapping functions. An example of this difficulty is illustrated by the *HSFA1* family in Arabidopsis which consists of four genes ( *HSFA1a* , *HSFA1b* , *HSFA1d* , and *HSFA1e* ). To uncover HSR phenotypes due to a loss of *HSFA1* function, at least three family members have to be knocked out as mutations in single or two *HSFA1* genes have no phenotypes in thermotolerance assays (Liu et al., 2011; Yoshida et al., 2011). Decreased mRNA levels of heat shock protein were documented in *hsfa1a/hsfa1b* double mutants, demonstrating that assaying gene expression can be used to overcome the sensitivity limitations of thermotolerance assays (Busch et al., 2005).

To overcome the limitations of conventional thermotolerance assays we built a system which would allow us to measure the HSR with high spatial and temporal resolution in large numbers of plants simultaneously. The system consists of an Arabidopsis reporter line containing the *HSP17.6* promoter driving a fast-folding GFP. *HSP17.6* (AT1G53540) encodes a small heat shock protein that is strongly induced by elevated temperatures and whose expression serves as a proxy for the HSR. The reporter line is imaged using a high-throughput automated fluorescence microscope that has a temperature controlled plant growth chamber (the RootScope), allowing 150 or more seedlings to be subjected to a heat shock while being imaged every four minutes (Kast et al., 2013).

We used the RootScope to investigate whether we could detect HSR expression phenotypes in a *HSFA1d* (AT1G32330) T-DNA mutant that does not have a thermotolerance phenotype in the absence of other mutations. The *hsfa1d-1* T-DNA line (SALK_022404) is a RNA null (Liu et al., 2011). Wild-type *HSP17.6:GFP* seedlings and *hsfa1d-1* mutants crossed into the reporter background were imaged during a one hour 37 ^o^ C heat shock and for 11 hours after the heat shock. Induction of *HSP17.6:GFP* expression was observed soon after the heat shock ended in both lines. Two differences became apparent during the recovery phase. First, the maximum *HSP17.6:GFP* expression level was lower in *hsfa1d-1* mutants than in wild type plants (1235±98 AU WT, 756±73 AU *hsfa1d-1* , t-test p-value<0.0001). Second, the time from the end of the heat shock until the expression peak was longer in *hsfa1d-1* mutants compared to wild type plants (220±36 minutes WT, 259±29 minutes *hsfa1d-1* , t-test p-value=0.0003) (Figure 1). The slower and smaller HSR in *hsfa1d-1* single mutants is consistent with previously established roles for *HSFA1* genes as positive regulators of the HSR (Liu et al., 2011; Yoshida et al., 2011).

Many Arabidopsis studies investigating temperature responses measure changes in gene expression immediately after a heat shock (Busch et al., 2005; Perez et al., 2009; Liu et al., 2011). This approach would not uncover a phenotype in the case of *hsfa1d-1* . The ability to uncover a gene expression kinetics phenotype highlights the power of measuring changes in gene expression over time as an approach for uncovering subtle quantitative phenotypes.

## Methods

The SALK_022404 T-DNA allele of *HSFA1d* was crossed to the previously described *HSP17.6:GFP* reporter line (Kast et al., 2013). F _1_ progeny were allowed to self-fertilize and F _2_ plants were screened for individuals homozygous for both the T-DNA insertion in *HSFA1d* and the *HSP17.6:GFP* reporter. The *HSFA1d* insertion was genotyped as described in Liu et al., 2011. The presence of two copies of the reporter was determined by screening F _3_ seeds using the RFP seed marker associated with the reporter which is in a pFAST-R07 backbone (Shimada et al., 2010; Kast et al., 2013). Families homozygous for the insertion and the reporter were assayed using the RootScope as previously described (Kast et al., 2013). Briefly, seeds were surface sterilized, plated on 0.5x MS media, and stratified at 4 ^o^ C for 48 hours. After stratification plates containing seedlings were transferred to E-30B growth chambers (Percival Scientific) and grown for four days at 22° under constant light conditions. The four day old seedlings were transferred to the RootScope’s temperature controlled imaging chamber and exposed to a one hour 37 ^o^ C heat shock followed by an 11 hour 22 ^o^ C recovery while on the microscope. HSP17.6:GFP fluorescence was measured in each plant root tip every four minutes.
